# Transcriptional and epigenetic responses to mating and aging in *Drosophila melanogaster*

**DOI:** 10.1186/1471-2164-15-927

**Published:** 2014-10-23

**Authors:** Shanshan Zhou, Trudy FC Mackay, Robert RH Anholt

**Affiliations:** Department of Biological Sciences, W. M. Keck Center for Behavioral Biology and Program in Genetics, Box 7614, North Carolina State University, Raleigh, NC 27695-7617 USA

**Keywords:** microRNA, Histone modification, ChipSeq, Environmental plasticity, Systems genetics

## Abstract

**Background:**

Phenotypic plasticity allows organisms to respond rapidly to changing environmental circumstances, and understanding its genomic basis can yield insights regarding the underlying genes and genetic networks affecting complex phenotypes. Female *Drosophila melanogaster* undergo dramatic physiological changes mediated by seminal fluid components transferred upon mating, including decreased longevity. Their physiological and behavioral effects have been well characterized, but little is known about resulting changes in regulation of gene expression or the extent to which mating-induced changes in gene expression are the same as those occurring during aging.

**Results:**

We assessed genome-wide mRNA, microRNA, and three common histone modifications implicated in gene activation for young and aged virgin and mated female *D. melanogaster* in a factorial design. We identified phenotypically plastic transcripts and epigenetic modifications associated with mating and aging. We used these data to derive phenotypically plastic regulatory networks associated with mating of young flies, and aging of virgin and mated flies. Many of the mRNAs, microRNAs and epigenetic modifications associated with mating of young flies also occur with age in virgin flies, which may reflect mating-induced accelerated aging. We functionally tested the plastic regulatory networks by overexpressing environmentally sensitive microRNAs. Overexpression resulted in altered expression of ~70% of candidate target genes, and in all cases affected oviposition.

**Conclusions:**

Our results implicate microRNAs as mediators of phenotypic plasticity associated with mating and provide a comprehensive documentation of the genomic and epigenomic changes that accompany mating- and aging-induced physiological changes in female *D. melanogaster*.

**Electronic supplementary material:**

The online version of this article (doi:10.1186/1471-2164-15-927) contains supplementary material, which is available to authorized users.

## Background

Phenotypic plasticity is the phenomenon whereby organisms with the same genotype can achieve different phenotypic endpoints in response to changes in external or internal environments. Phenotypic plasticity thus allows organisms to respond rapidly to changing environmental circumstances. Understanding the genomic basis of phenotypic plasticity can yield insights regarding the underlying genes and genetic networks affecting complex phenotypes, since mutations in genes whose expression changes in different environments also affect the phenotype [1–5].

Most previous studies of genome wide transcriptional responses to environmental perturbations have utilized microarray technology, which has a lower dynamic range than direct sequencing of mRNAs and can be influenced by DNA polymorphisms between the target genotype and the reference genotype used to design the array. We used whole genome RNA sequencing to alleviate these limitations and included a comprehensive analysis of microRNAs (miRNAs). MicroRNAs bind to the 3′ untranslated regions of mRNA, preventing its translation, polyadenylation or stability, and play essential roles in signaling pathways that regulate development and differentiation [6, 7]. Although miRNAs have been implicated in lifespan determination in *Caenorhabditis elegans* and *Drosophila* [8, 9], little is known regarding their role in phenotypic plasticity. Finally, epigenetic modifications have been proposed as an important aspect of phenotypic plasticity through their regulation of gene expression [10–12]. However, we know little of the complex relationships between genome-wide transcriptional responses to environmental perturbations and their regulation by miRNAs and epigenetic modifications.

Here, we utilized next generation sequencing technology to assess the plastic responses of mRNAs, miRNAs and three major histone modifications (H3K4me1, H3K4me3, and H3K9ac) to mating and aging in *D. melanogaster* females, in a factorial design. Mating and aging have previously been shown to elicit transcriptional responses in *Drosophila* [4, 9, 13–20]. During mating, male *D. melanogaster* transfer a complex mixture of accessory gland derived peptides and proteins together with sperm into the female reproductive tract. These peptides exert profound physiological and behavioral changes in the female which are thought to enhance the male’s competitive reproductive success [21–23]. They activate oocyte development and accelerate egg laying, reduce receptivity of the female to additional matings, and enhance the female’s immune competence [21–23]. In addition, mated females have a reduced lifespan compared to virgin females [4, 24]. The effects of several accessory gland peptides have been well characterized, including ovulin [25] and the sex peptide [26]. The latter exerts its effect by binding to receptors that are expressed in the reproductive tract and in the central nervous system [26]. While the abundances of many hundreds of transcripts are known to change with age [2, 8, 9, 14–16, 20, 27], it is not known to what extent these patterns vary between virgin and mated flies and whether the reduced longevity of mated females reflects an acceleration of normal senescence. We used our data to construct interaction networks between phenotypically plastic miRNAs, their target genes and associated histone 3 markers. We functionally validated the effects of miRNAs on oviposition in mated females to demonstrate that miRNAs are instrumental in facilitating post-mating physiological and behavioral changes in female *D. melanogaster*.

## Results

We assessed changes in genome wide mRNA, miRNA and epigenetic modifications for 3–5 day old and 4-week old virgin Canton S (B) [28] *D. melanogaster* females and females which were maintained in the continuous presence of males with multiple opportunities for mating. We refer to the latter as “mated” females. We evaluated overall differences in transcriptional and epigenetic responses to mating and to aging, as well as differences in the molecular signatures of plasticity between young and old virgin and mated flies, and mated and virgin young and old flies. This design enables us to assess whether or to what extent mating induces changes in molecular response profiles that would otherwise occur later in life.

### mRNA plasticity

We obtained mRNA expression profiles for each of the four physiological conditions by RNA sequencing. An average 95.7% of sequences had quality scores higher than 37.65, with over 65× coverage of the estimated 30 Mb *Drosophila* transcriptome. We detected 26,151 mRNA sequences, including 15,610 annotated transcripts and 10,541 previously unannotated transcripts. Some of the unannotated transcripts could be artifacts of mapping short reads corresponding to premature unspliced mRNAs back to the genome; others represent novel intergenic long non-coding RNAs. We identified 647 unique genes that were differentially expressed post-mating or as a function of age. These include 344 genes with predicted transcripts of unknown function, 23 annotated non-coding transcripts and two novel (i.e., previously unannotated) genes. The remaining 278 genes included multiple members of gene families, such as the *IM*, *Jonah*, *Cp, Cpr, Twdl, Osi, Vm, Mal* and *Obp* families (Additional file 1). Among the differentially expressed genes, 474 showed altered transcript levels in young flies after mating; 160 showed altered transcript levels between young and old virgin flies; and 279 genes showed altered expression between young and old mated flies (Additional file 1). There was modest overlap among these categories (Additional file 2: Figure S1). Only 18 differentially expressed transcripts were identified between aged virgin and mated flies. This suggests that the largest post-mating differences in transcript profiles are observed in young females and these differences fade as flies age.

**Figure 1:**
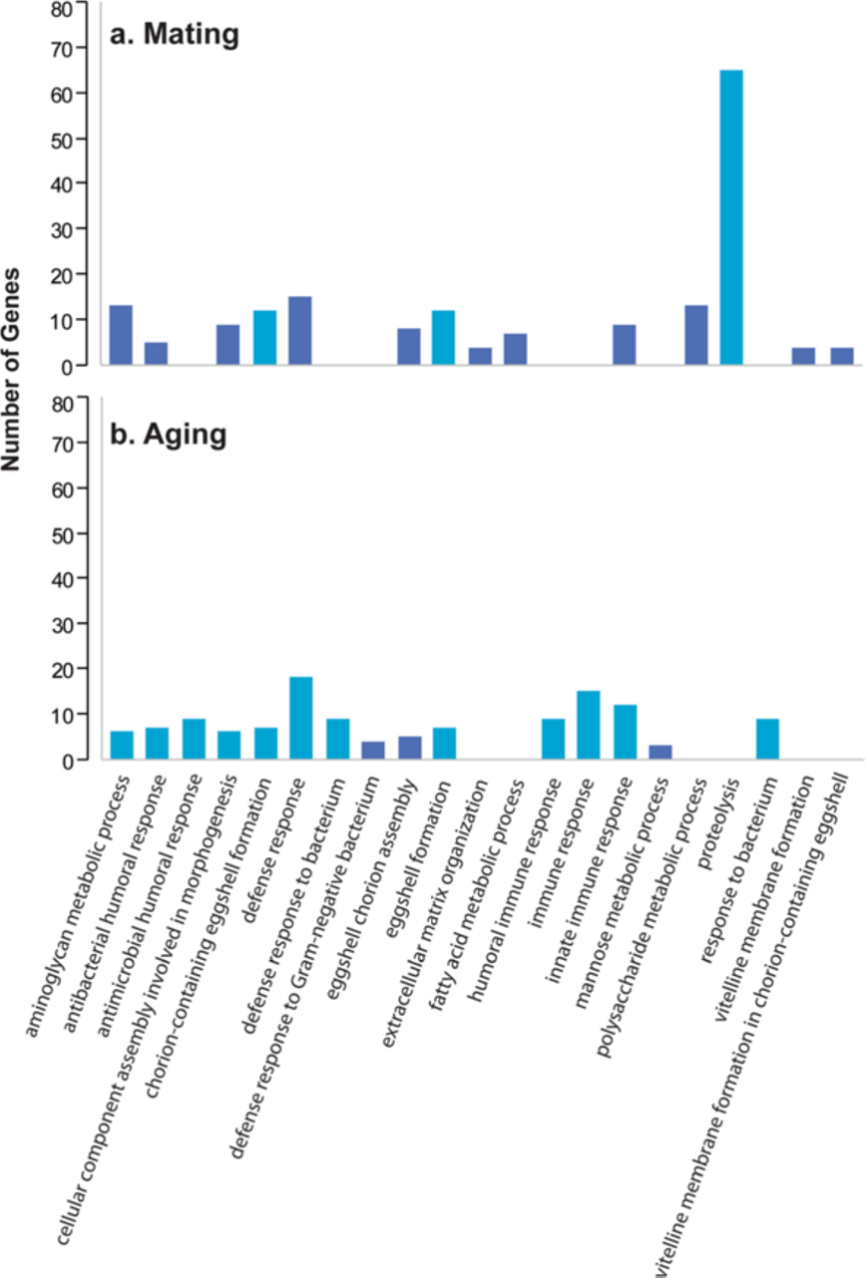
**Gene ontology enrichment of transcripts with altered expression as a result of mating or aging.** Gene ontology enrichment was assessed for transcripts that changed expression levels in young flies before and after mating **(a)** and in young *vs* aged virgin flies **(b)** Light blue bars exceed the Benjamini corrected threshold of *P* <0.05.

We performed Gene Ontology (GO) enrichment analyses for the two treatments with the largest numbers of environmentally responsive transcripts: the plastic response to mating for young flies (Figure 1a) and the plastic response to aging of virgin flies (Figure 1b). Transcripts with altered expression levels following mating show strong enrichment of GO terms associated with egg development and proteolysis, in agreement with a previous study [13]; as well as GO terms associated with bacterial defense and immune responses (Figure 1a). GO categories associated with immune/defense response, egg shell formation and metabolism were enriched in the comparison between young and old virgin flies (Figure 1b), also in agreement with previous studies [14–16]. There is partial concordance between GO categories between the transcript profiles of young flies before and after mating and young and aged virgin flies, indicating that some biological processes undergo specific post-mating or aging-related changes in transcript abundance levels, whereas others reflect post-mating changes that would occur later in the lifespan in the absence of mating.

### miRNA plasticity

**Figure 2:**
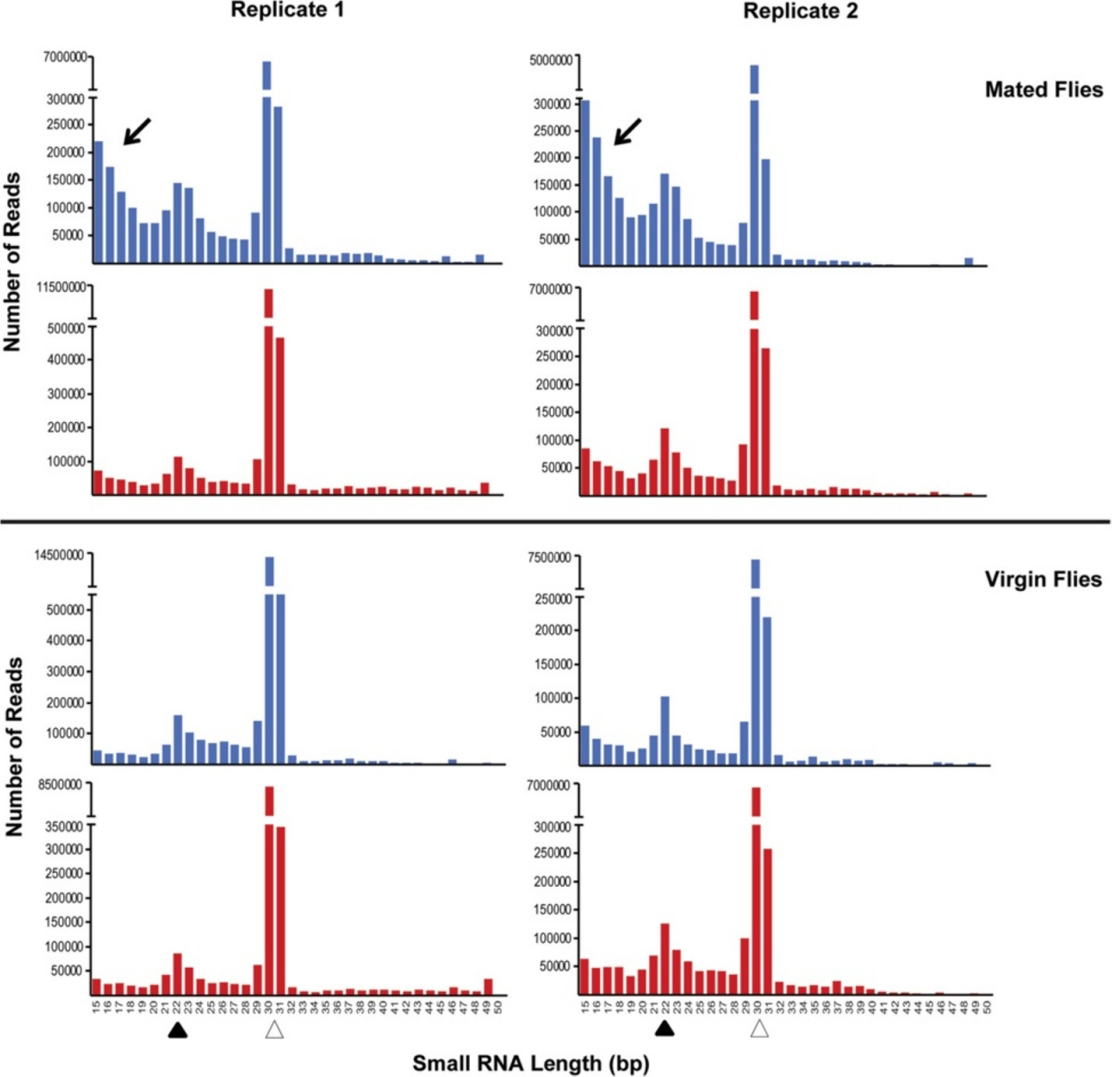
**Size distributions of small RNAs in young (blue) and aged (red) virgin and mated flies.** Black and open arrowheads indicate the regions corresponding to miRNAs and rasiRNAs, respectively. The arrows point at small fragments apparent only in young flies after mating that likely reflect mRNA degradation products generated during the maternal to zygotic transition.

We extracted and sequenced small RNAs (<200 nt, including miRNAs) from the same samples used to assess genomic responses of mRNAs to mating and aging. Examination of the distribution profiles of small RNAs showed prominent peaks at 22 bp and 30 bp, corresponding to miRNAs and rasiRNAs (the *Drosophila* equivalent of piRNAs), respectively (Figure 2). A prominent peak of small RNA fragments around 15 bp is evident in replicate samples of flies following mating. These fragments are transient and are not observed for older mated flies, nor are they observed in young or old virgin flies. These small mRNA fragments are likely due to degradation of maternal RNA in embryos during the maternal to zygotic transition following fertilization [29, 30]. We mapped the 15-18 bp RNA fragments across all samples to the genome sequence and identified 147 genes that give rise to degradation fragments exclusively in young flies after mating (Additional file 3: Table S1). Among these genes, 64 encode predicted transcripts of unknown function. These genes also comprise genes associated with developmental processes and, intriguingly, four genes encoding chemoreceptors (*Gr39b*, *Gr93d*, *Or13a*, *Or45a*).

**Figure 3:**
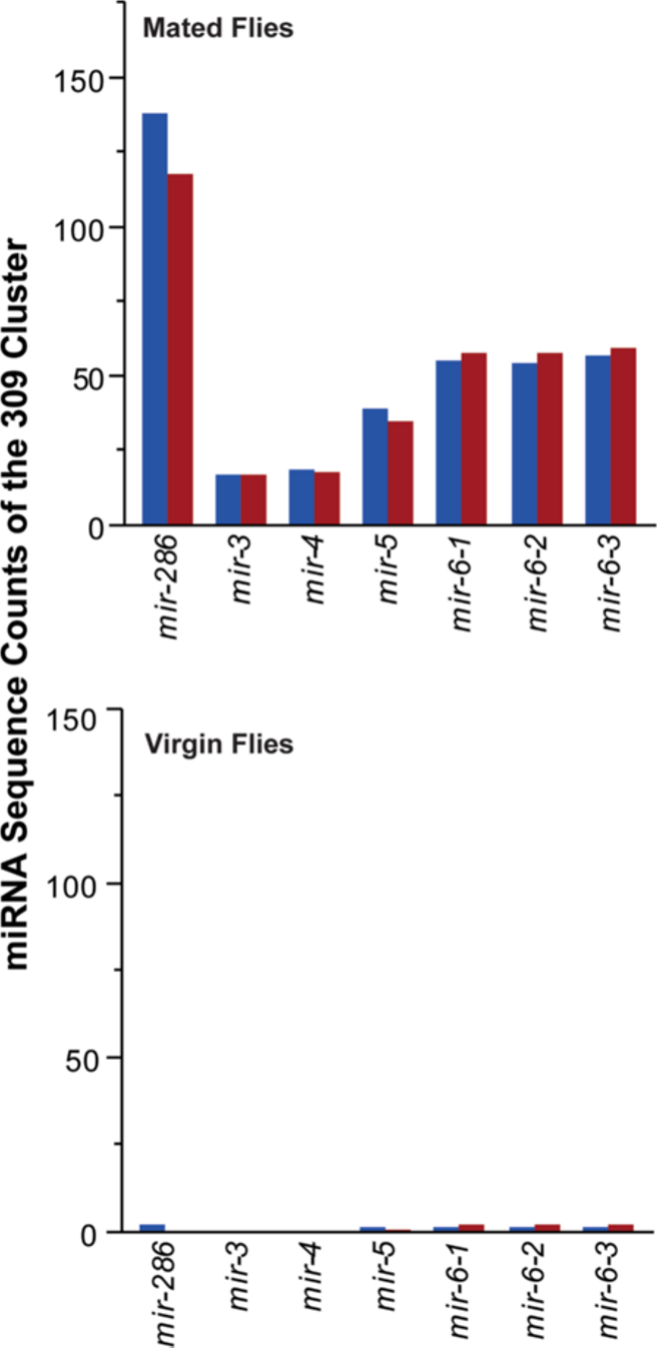
**Changes in mir-309 cluster expression after mating in young (blue) and old (red) flies.**

We detected 149 mature miRNAs from 105 precursors, of which 41 miRNAs from 39 precursors with 28 conserved seeds showed significant differences across the four conditions (Additional file 4). Similar numbers of miRNAs show plastic responses to the four treatments. We find 27 differentially regulated miRNAs after mating in young flies and 22 in old flies; and 17 miRNAs differentially regulated with aging in virgin flies and 28 in mated flies, with considerable overlap in plastic responses of miRNAs among the four physiological conditions (Additional file 5: Figure S2). Of particular interest is the miRNA 309 cluster, which, in addition to *mir-309*, comprises *mir-286*, *mir-3*, *mir-4*, *mir-5* and *mir-6*. The latter gives rise to three alternative stem-loop configurations with similar conserved seed regions [31, 32]. Zygotic expression of these miRNAs has been associated with degradation of maternal mRNA in embryos [29, 32]. Indeed, these miRNAs are only expressed in mated flies and are virtually absent in virgins (Figure 3). Among the 147 genes that are uniquely implicated in maternal degradation, 45 contain 21 conserved binding sites for miRNAs that are differentially expressed after mating, and 18 genes are targets of the miRNA 309 cluster.

### Epigenetic plasticity

Epigenetic regulation in *D. melanogaster* occurs primarily through association of modified histones with target DNA sequences [33]. Numerous post-translational modifications of histone proteins have been identified, including methylation, acetylation, phosphorylation, ubiquitylation, SUMOylation, biotinylation and ADP-ribosylation [11, 34, 35]. Some histone modifications act in *cis* to change chromatin structure directly, whereas others act in *trans* to affect the recruitment of a protein complex that regulates gene expression [36]. Modified histone protein residues can serve as docking sites for transcription factors and other chromatin-modifying enzymes to regulate transcription or induce further chromatin remodeling [11, 12, 34, 35]. We examined three common histone 3 modifications (H3K4me1, H3K4me3, H3K9ac) previously implicated in gene activation [36].

We used immuno-coprecipitation to identify DNA segments differentially associated with each of the histone 3 modifications across the genome. We identified 3,484, 5,467, and 4,986 peaks of co-precipitated DNA fragments for H3K4me1, H3K4me3 and H3K9ac, respectively; of which 710 (H3K4me1), 1,513 (H3K4me3) and 1,819 (H3K9ac) co-precipitated fragments were differentially expressed among the four physiological conditions (Additional file 6: Figure S3). We found similar numbers of up- and down-regulated genes for each variable histone modification (Table 1; Additional files 7, 8 and 9). The number of histone modifications associated with aging was larger than those associated with post-mating changes, especially for H3K4me3 (Table 1). We identified candidate histone regulated genes associated with co-precipitated fragments by applying a 2 kb window downstream and upstream of each gene. On average, 3.3 candidate genes were associated with each histone peak (Table 1), of which ~24% are novel transcripts.

**Table 1:** Number of genes associated with changes in histone modifications*

Histone modification	Post-mating changes in young flies		Post-mating changes in aged flies		Changes associated with aging in virgin flies		Changes associated with aging in mated flies	
	Histone peaks	Genes	Histone peaks	Genes	Histone peaks	Genes	Histone peaks	Genes
**H3K4me1**								
*Up-regulated*	178	536	259	847	253	750	284	911
*Down-regulated*	205	677	249	772	291	971	299	1002
**H3K4me3**								
*Up-regulated*	281	959	530	1899	611	2000	703	2482
*Down-regulated*	383	1293	482	1708	693	2382	710	2534
**H3K9ac**								
*Up-regulated*	512	1618	427	1427	816	2376	806	2598
*Down-regulated*	536	1680	458	1428	823	2553	834	2755

*Differences between conditions were estimated by subtracting histone peak amplitudes in virgin flies from those in mated flies and in young flies from those in aged flies. Only histone modification peaks that are significantly variable across the four conditions (FDR <0.05) with differences that are larger than one pooled standard deviation are counted.

About 10% of differentially modified fragments after mating in young flies were shared between H3K4me1 and H3K4me3, and between H3K4me1 and H3K9ac, while about 15% were shared among H3K4me3 and H3K9ac, and only about 1% of these fragments were associated with all three modified histone marks (Additional file 10: Figure S4). Similarly, about 17% of differentially modified fragments identified as a function of aging of virgin females were shared between H3K4me1 and H3K4me3, and between H3K4me1 and H3K9ac, while as much as 36% of precipitated DNA sequences were shared among H3K4me3 and H3K9ac, and about 5% of sequences were associated with all three modified histone marks (Additional file 10: Figure S4).

We asked to what extent histone modifications induced by mating are permanent throughout the lifespan of reproductively active females. We found that 18%, 9% and 11% of H3K4me1, H3K4me3 and H3K9ac modifications, respectively, that occurred post-mating in young flies persisted at 4 weeks of age when females were maintained in the presence of males. Next, we asked how many of these histone modifications also occur as a result of aging in the absence of mating. We found that 65%, 73% and 77% of these H3K4me1, H3K4me3 and H3K9ac modifications, respectively, ultimately occur in virgin flies, but only at later age. These markers, therefore, represent epigenetic mating-induced accelerated aging changes and are associated with up to 539 unique candidate genes, of which 421 are annotated (Additional file 11). These include genes associated with oocyte differentiation, including *stonewall*, *14-3-3zeta, bazooka, missing oocyte,* and *egalitarian.* Histone modifications that affect these candidate genes may reflect persistent post-mating stimulation of oogenesis.

To assess to what extent environmentally plastic transcripts might be causally associated with epigenetic regulation we asked which putative histone regulated genes showed altered transcriptional regulation across the four experimental conditions (Table 2). The majority of genes with altered transcriptional regulation corresponded with candidate target genes for the three histone marks when young virgin flies are compared to young mated flies and when young mated flies are compared to aged mated flies (Table 2). Many fewer genes with altered transcriptional regulation corresponded with candidate target genes when young virgin flies were compared to aged virgins and when old virgin flies were compared to old mated flies (Table 2). Although these proportions roughly parallel differences in the numbers of transcripts with altered abundance levels as a consequence of mating or aging, they illustrate that different transcripts interact with histone 3 upon mating and as a function of age. Among genes with altered transcriptional regulation that interact with modified histone 3, only a few are associated with more than one histone mark, indicating target specific interactions for each histone modification with these genes (Table 2).

**Table 2:** Numbers of candidate histone regulated genes with altered expression after mating or upon aging*

Comparison	H3K4me1		H3K4me3		H3K9ac	
	Up-regulated	Down-regulated	Up-regulated	Down-regulated	Up-regulated	Down-regulated
Young flies						
Virgin vs. Mated	13	13	12	15	19	20
Virgin flies						
Young vs. Aged	4	3	4	5	2	4
Mated flies						
Young vs. Aged	5	6	24	18	16	11
Aged flies						
Virgin vs. Mated	1	0	0	0	0	0

*Numbers in the table represent genes associated with histone marks. *Up-regulated* and *Down-regulated* refer to the histone marks detected by immuno co-precipitation. Differences between conditions were estimated by subtracting histone peak amplitudes in virgin flies from those in mated flies and in young flies from those in aged flies.

### Phenotypically plastic regulatory networks

We identified target genes that undergo changes in expression for all miRNAs with altered transcriptional regulation, and recruited genes known to interact with these target genes from the Drosophila Interactions Database [37] to obtain integrative networks that provide a comprehensive visualization of post-mating or aging-associated changes in whole genome transcriptional profiles, and superimposed histone marks on the networks. We only included genes with more than one interaction with miRNA target genes. Since very few transcripts were altered in the comparison of old virgin and mated flies, we derived these plastic regulatory networks for three conditions: young virgin and mated flies, young and old mated flies, and young and old virgin flies.

**Figure 4:**
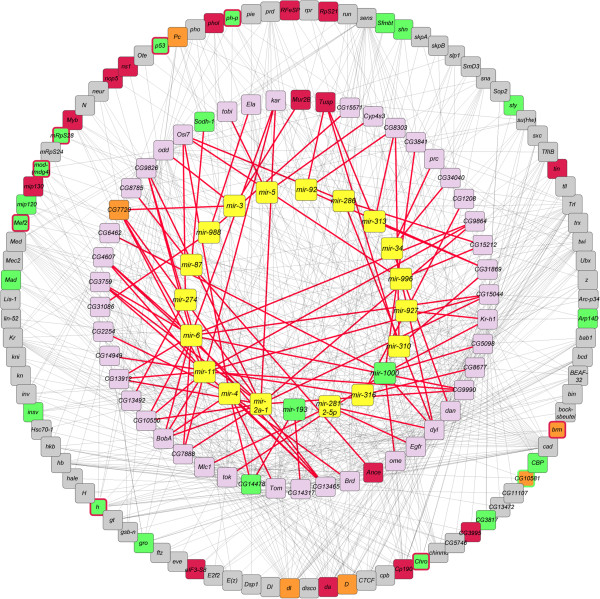
**A network of miRNAs, their target genes, and potential interacting partners associated with post-mating changes.** miRNAs that change expression in young flies after mating are shown in the inner circle in yellow boxes and are connected to their target genes, shown in the middle circle, with red lines. The miRNA target genes are connected with known interaction partners, shown in the outer circle, with grey lines. Genes that are targets for H3K4me1, H3K4me3 and H3K9ac, are shown in orange, green and red boxes, respectively. Targets for two histone 3 marks are indicated with an additional border color. Note that only miRNAs and target genes that undergo altered transcriptional regulation are included in the network, and only genes interacting with more than one miRNA target are represented in the outer circle.

The network associated with post-mating changes in young flies comprised 46 target genes for 20 miRNAs, and these target genes have 92 interacting partners (Figure 4; Additional file 12). In addition to genes that encode predicted transcripts of unknown function, developmental genes feature prominently among the latter. The network reveals several notable features. First, there are shared targets for multiple miRNAs. Second, these target genes interact with multiple partners and each interacting partner interacts with multiple miRNA targets, thus forming a collage of interacting ensembles that are embedded in the network. Third, 13% of miRNA target genes and 37% of their interacting partners are targets for regulation through histone modifications. In addition, two miRNAs, *mir-193* and *mir-1000*, are themselves potential targets for H3K4me3. It is reasonable to infer from the network structure that regulatory modifications at a limited number of focal genes can affect the expression of a suite of interacting partners.

**Figure 5:**
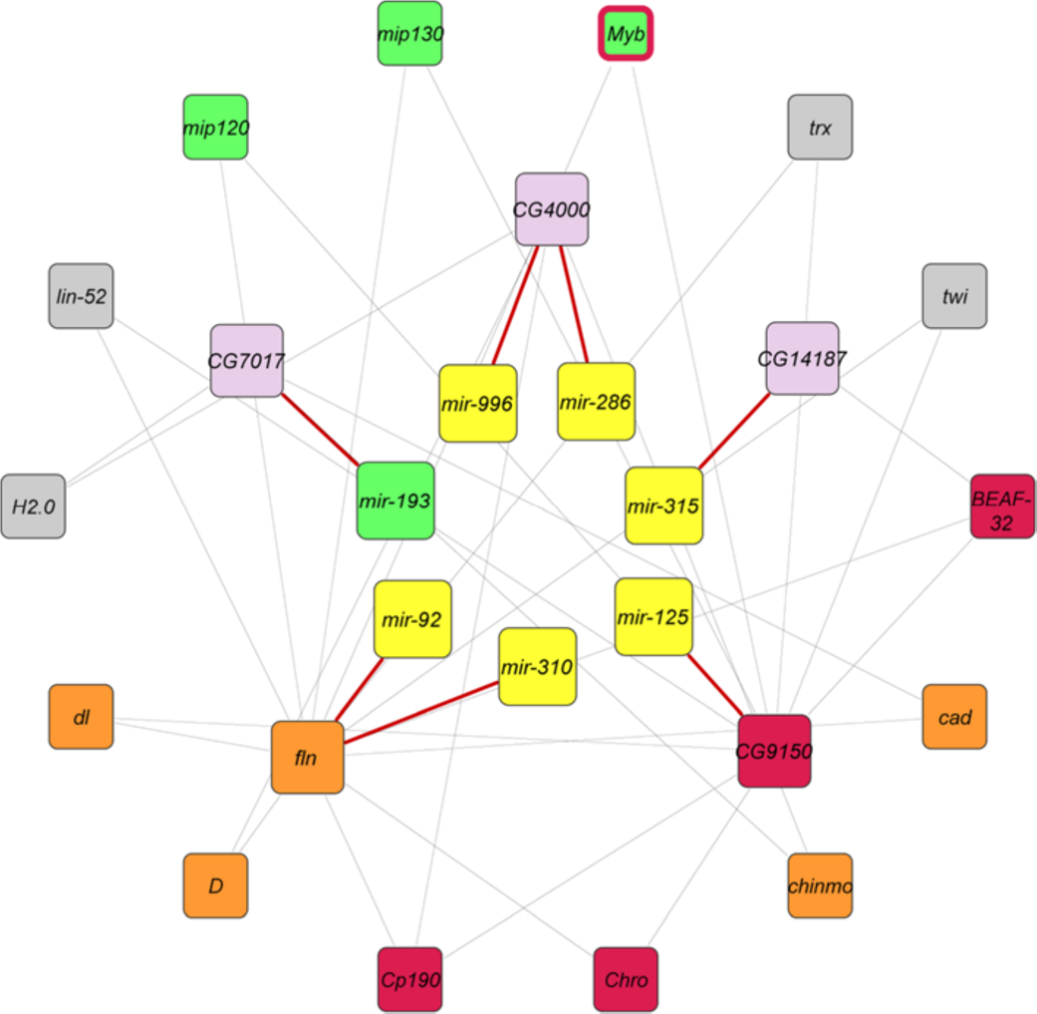
**A network of miRNAs, their target genes, and potential interacting partners associated with aging.** miRNAs that change expression upon aging of virgin flies are shown in the inner circle in yellow boxes and are connected to their target genes, shown in the middle circle, with red lines. The miRNA target genes are connected with known interaction partners, shown in the outer circle, with grey lines. Genes that are targets for H3K4me1, H3K4me3 and H3K9ac, are shown in orange, green and red boxes, respectively. *Myb* is a target for both H3K4me3 and H3K9ac, as indicated by the red border around the green box. Note that only miRNAs and target genes that undergo altered transcriptional regulation are included in the network, and only genes interacting with more than one miRNA target are represented in the outer circle.

A similar analysis of transcriptional changes between young and old virgin flies gives rise to a smaller network, comprised of only seven miRNAs, five target genes, and 14 genes interacting with these targets (Figure 5; Additional file 12). Of the latter, 10 are potential targets for regulation by modified histone 3, as are two of the miRNA target genes. Among the seven miRNAs, five overlap with the network associated with post-mating changes, including *mir-193*. It should be noted that in this case the composition of differentially expressed miRNA target genes restricts connectivity, which limits the size of the network. Nevertheless, it is clear that the genetic architectures that underlie changes in gene expression are distinct with regard to post-mating changes and aging, as might be expected.

**Figure 6:**
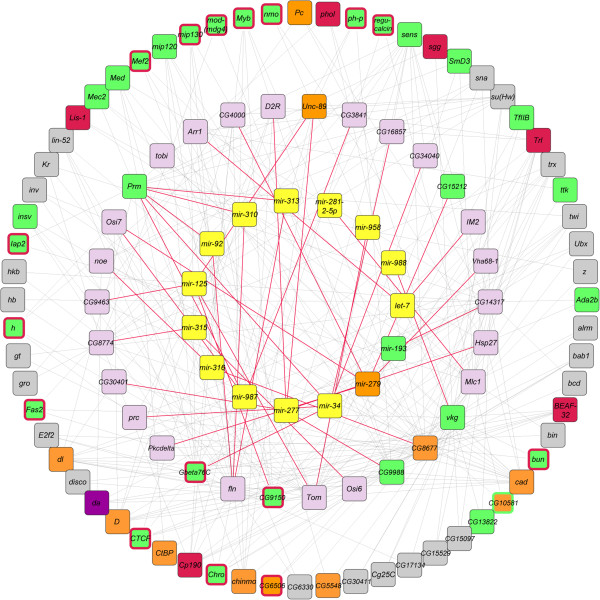
**miRNAs, their target genes, and potential interacting partners associated with aging and mating.** miRNAs that change expression after aging are shown in the inner circle in yellow boxes and are connected to their target genes, shown in the middle circle, with red lines. The miRNA target genes are connected with known interaction partners, shown in the outer circle, with grey lines. Genes that are targets for H3K4me1, H3K4me3 and H3K9ac, are shown in orange, green and red boxes, respectively. Targets for two histone 3 marks are indicated with an additional border color. Note that only miRNAs and target genes that undergo altered transcriptional regulation are included in the network, and only genes interacting with more than one miRNA target are represented in the outer circle.

The network associated with aging in mated flies comprises 15 miRNAs, 27 miRNA target genes and 64 interacting partners (Figure 6; Additional file 12). Two miRNAs, *mir-193* and *mir*-*279* are potential targets of H3K4me3 and H3K4me1, respectively. Eight miRNA target genes (~29%) and 39 (~60%) interacting partners are potentially regulated by histone modifications.

### Effects of micro-RNA overexpression on target gene expression

To test the connectivity of the computational networks (Figures 4, 5 and 6) and evaluate causality, we overexpressed six miRNAs for which overexpression lines were available (*mir-281-1*, *mir-286*, *mir-34*, *mir-92b, mir-310* and *mir-988*) using the binary *GAL4-UAS* expression system [38] under a universal ubiquitin promoter. Evidence that the driver was effective comes from the observation that no viable offspring could be obtained when *mir-281-1* and *mir-310* were overexpressed. The remaining overexpression lines generated F1 progeny, in which we used quantitative RT-PCR to monitor expression of target genes in virgin and mated flies contemporaneously with F1 progeny obtained by crossing the control progenitor strain to the *ubiquitin-GAL4* driver line. We assessed whether changes in their corresponding target genes that were differentially expressed between young virgin and mated females were significantly different after mating in control lines compared to the miRNA overexpression lines (Student’s *t*-test, *P* <0.05). Despite differences in genetic backgrounds between the *GAL4-UAS* offspring and the original Canton S (B) line used to derive the networks, we observed significant effects for 13 of the 20 target genes tested (70%; Table 3). Thus, perturbation of miRNAs results in disruption of target gene expression that occurs during changes in physiological state and directly implicates miRNAs in the regulation of post-mating and aging-dependent gene expression.

**Table 3:** Real time PCR confirmation of interactions between miRNAs and target genes*

miRNA	Target gene	Expression in virgin vs. mated female flies ( ***P-***Value)		
		Control cross	miRNA overexpression cross	Effect on target gene expression
*mir-286*	*CG15212*	0.498	0.037	+
	*CG4000*	0.033	0.064	+
	*dyl*	0.241	0.018	+
	*Osi7*	0.380	0.006	+
	*Pkcdelta*	0.044	0.364	+
	*Vha68-1*	0.013	0.348	+
*mir-34*	*CG16857*	0.034	0.434	+
	*CG34040*	0.046	0.036	-
	*dan*	0.204	0.367	-
	*Gbeta76c*	0.277	0.010	+
	*prc*	0.240	0.410	-
	*prm*	0.060	0.441	-
*mir-92b*	*CG31869*	0.351	0.008	+
	*CG8303*	0.446	0.026	+
	*fln*	0.378	0.034	+
	*Tusp*	0.075	0.032	+
*mir-988*	*CG15571*	0.038	0.226	+
	*CG31086*	0.036	0.417	+
	*Ela*	0.240	0.139	-
	*vkg*	0.143	0.058	-

*Student’s *t*-tests were used to calculate *P*-values of significant differences between expression in virgin and mated flies. Overexpression of a miRNA has an effect on the target gene when differential expression of the target gene is only significant (*P* <0.05) in control females, but not in the miRNA overexpressing females, or *vice versa*.

### Effects of micro-RNA overexpression on egg laying

**Figure 7:**
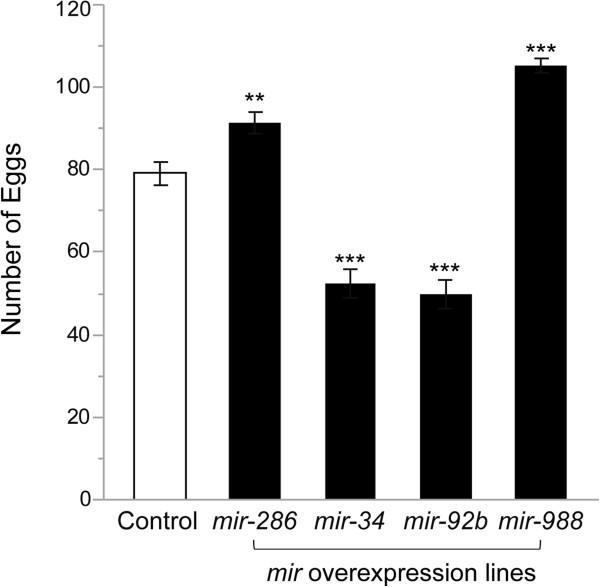
**Effects of miRNA overexpression on egg laying by mated females.** Number of eggs laid by five young mated females over 18 hours between control and *mir-286*, *mir-34*, *mir-92b* and *mir-988* overexpression lines. Two stars indicate *P* <0.01 and three stars indicate *P* <0.0001. Error bar shows standard error.

Stimulation of oogenesis and oviposition is the most profound change induced in females as a consequence of mating [21]. To assess causality between post-mating modulation of miRNA expression and oviposition, we measured egg laying after mating by control females and females overexpressing *mir-286*, *mir-34*, *mir-92b*, or *mir-988*. Mating causes a reduction in expression of *mir-34*, *mir-92b* and *mir-988* and an increase in expression of *mir-286* (Figure 3; Additional file 4). Females that overexpress *mir-34* and *mir-92b* lay 34% and 37% fewer eggs after mating, whereas females that overexpress *mir-286* and *mir-988* lay 16% and 33% more eggs than controls (Figure 7). Thus, post-mating changes in miRNA expression levels are causally associated with physiological changes.

## Discussion

We identified genomic and epigenomic changes in gene expression that are associated with mating and aging in female *D. melanogaster*. We found that 26 miRNAs change expression levels after mating, including transient expression of members of the *mir-309* cluster associated with the maternal to zygotic transition that results in maternal mRNA breakdown [29, 30]. The *mir-309* cluster is analogous to *mir-430*, which mediates the maternal to zygotic transition in zebrafish [39]. Up-regulation of the *mir-309* cluster after mating is accompanied by the transient accumulation of small RNA fragments, likely representing breakdown products of degraded maternal RNAs in embryos. In *D. melanogaster*, loci required for the destabilization of distinct subsets of maternal mRNAs during the maternal to zygotic transition have been mapped to different chromosome arms [40], indicating that different mechanisms may mediate maternal RNA degeneration in the zygote. The appearance of RNA fragments only in young flies after mating could be due to subsequent changes that may include epigenetic modifications, which increase the efficiency of the removal of such fragments during repeated matings.

We identified target genes for miRNAs that change transcript abundance levels after mating. We examined similar changes in genome-wide expression levels of miRNAs and their associated target genes that occur as females age. In addition, we analyzed epigenetic modifications mediated via histone 3 that are associated with mating and aging. We were able to construct integrative genetic networks from a subset of genes with altered expression due to mating or aging to visualize part of the genetic architectures that underlie these physiological changes. We found that the largest differences in transcript profiles occur in young females after mating. Although the genetic architectures that underlie changes in gene expression are distinct with regard to post-mating changes and aging, we found epigenetic modifications that occur post-mating in young flies and at later age in virgin flies, consistent with mating-induced accelerated aging and consequent shortening of the lifespan of mated females [24] (Additional file 3).

The power of our analysis is limited by the small number of replicate samples and histone modifications analyzed, and possible differences in genomic responses in different genetic backgrounds. Thus, the complement of genes that undergo altered regulation as a consequence of physiological state and epigenetic modifications that could regulate their expression provides only a partial view of the genetic architectures that regulate physiological state-induced regulation of gene expression. Furthermore, genes associated with histone marks are commonly assigned based on chromosomal location [36, 41, 42]. Demonstrating causal relationships with candidate genes within such chromosomal regions remains challenging, and it is difficult to unambiguously identify false positives or exclude long-distance effects of modified histones on gene expression. We alleviated this concern to some extent by focusing on histone modifications associated with co-localized genes that undergo altered expression. We also note that our analyses were performed on whole flies and we cannot exclude that some changes in miRNA expression or histone modifications may be compartmentalized to certain tissues.

A large proportion of genes with changes in transcript abundance are members of gene families. Members of the *Jonah*, *Cp*, *Twdl,* and *Mal* gene families show antagonistic changes in transcript abundances after mating and as a result of aging, whereas *Vm* family members show changes in the same direction. Similarly, some genes with altered expression that belong to gene ontology categories shared between mating and aging conditions also show antagonistic effects. We speculate that these antagonistic changes may be part of the molecular machinery that mediates trade-offs between reproduction and lifespan [24]. In addition to restricted expression in a limited number of cells, antagonistic regulation in different tissues could also be a limiting factor in detecting genes that undergo altered transcriptional regulation (*e.g.* we did not detect altered transcript abundance of *smaug,* which triggers the maternal to zygotic degradation [30, 43]).We observed many histone modifications after mating or upon aging that are not accompanied by changes in corresponding transcript abundances. Here, the target gene might be remote from the histone-DNA interaction site. Alternatively, interaction between the histone and the target gene might prevent activation of this gene at a later stage or inactivation of a gene that is actively expressed. Furthermore, the networks centered on miRNAs and their target genes (Figures 4, 5 and 6) do not include all transcripts with altered expression levels, nor do they include all potential target genes for epigenetic regulation. Nevertheless, the complex structure of these networks raises the possibility that epigenetic regulation of target genes for histone 3 could also indirectly affect the expression of interconnected genes.

In addition to epigenetic modifications that uniquely occur after mating, we identified identical epigenetic markers that occur also in virgin females at advanced age. These mark post-mating changes that relate to the shortening of female lifespan after mating, possibly due to the increased energy expenditure required for egg production [21, 24].

Overexpression of miRNAs confirmed the biological relevance of the derived environmentally plastic regulatory networks. Despite differences in genetic backgrounds, altered expression of miRNAs correlated with altered expression of ~70% of their candidate target genes, and in all cases miRNA overexpression affected post-mating oviposition. Although we analyzed only a subset of miRNAs for which viable overexpression lines could be obtained, it is reasonable to assume that these observations would extend to the larger ensemble of miRNAs.

Since this study focused on whole flies, it remains to be determined to what extent the genomic and epigenomic changes we have observed are compartmentalized to reproductive organs or the central nervous system. Previous studies have implicated steroid hormones in regulating expression of miRNAs [44, 45]. Odorant binding proteins expressed in the male accessory gland could conceivably serve as carriers for such ligands [19]. However, the precise mechanisms by which signals transmitted during mating regulate gene expression remain to be explored. Nevertheless, this study presents to the best of our knowledge the first demonstration that miRNAs play a critical role in mediating post-mating changes in female flies and a comprehensive documentation of the genomic and epigenomic changes that accompany mating-induced physiological changes as well as aging in female *D. melanogaster*.

## Conclusions

To gain insight regarding the genes and regulatory networks underpinning plastic changes after mating and during aging, we identified mRNAs, microRNAs and epigenetic modifications with significantly different expression after mating or as a function of aging. We used these data to derive phenotypically plastic regulatory networks centered on environmentally sensitive microRNAs associated with aging and mating, and identified several biomarkers of mating-induced accelerated aging. Overexpression of several plastic microRNAs resulted in altered expression of candidate target genes and affected oviposition. MicroRNAs are thus critical in mediating post-mating changes in gene expression. These data provide comprehensive documentation of the genomic and epigenomic changes that accompany mating- and aging-induced physiological changes in female *D. melanogaster*.

## Methods

### *Drosophila*stocks

Isogenic Canton S (B) flies [28] were reared on cornmeal-molasses-yeast medium at 25°C under a 12 hour light–dark cycle. Adults were collected immediately after eclosion and subjected to two treatments. In the first treatment, adults were allowed to mate at a density of 25 females and 25 males per vial. In the second treatment, 50 females were placed in each vial and maintained as virgins. Flies were transferred to new vials every 2 days. Female flies were collected at 3–5 days (young flies) or at 4 weeks (aged flies) at the same time of day (1 pm-3 pm) and flash frozen on dry ice.

### mRNA and miRNA sequencing

Two independent samples of 25 female flies were used for each condition (young virgin flies, young mated flies, aged virgin flies and aged mated flies). RNA was extracted and separated into a small RNA (<200 nts) and large RNA fraction (>200 nts) using the Qiagen miRNeasy kit. rRNA was depleted from the large RNA fraction using Ribo-Zero rRNA Removal Kits (Epicentre, Inc.) to enrich for mRNA. We used enriched mRNA to prepare bar-coded libraries. Four mRNA samples were pooled in equal molarity from each replicate and sequenced in one 50 bp single read Illumina HiSeq2000 lane. Small RNA libraries were prepared using the NEXflex Small RNA Sequencing kit (Bioo, Inc.) and enriched for miRNA, siRNA and piRNA following gel electrophoresis. All 8 bar-coded samples were pooled in equal molarities and sequenced in one 50 bp single read Illumina HiSeq2000 lane.

mRNA sequences were aligned and assembled using Bowtie2-2.0.6, Tophat-2.0.7 and Cufflinks-2.0.2 [46, 47]. We used Cuffdiff to analyze pairwise differential expression between the conditions of transcripts, genes, splicing and promoter uses [48]. We first trimmed small RNA sequences to remove the adapter sequence before aligning them to the Drosophila miRBase [49] using miRExpress [50]. We then used quantile normalized counts for differential analysis implemented by the R package (lmPerm) using permutation with a one-way ANOVA model, *Y* = *μ* + *C* + *ϵ,* where *μ* is the overall mean, *C* designates condition and *ϵ* the error term. We used FDR to correct for multiple-testing under dependency [51]. One pooled standard deviation was used for *post hoc* pairwise comparisons. We identified putative miRNA targets using the TargetScan Fly [52] and MinoTar data bases [53]. Raw data can be accessed in the National Center for Biotechnology Information Sequence Read Archive (SRA) under accession number SRP048388.

### Chromatin immunoprecipitation and ChIP sequencing

Two independent samples of 400 female flies were used for each condition. Flies were ground in liquid nitrogen. Samples were then cross-linked with 1% formaldehyde for 10 min and lysed, and chromatin was digested into mono- and di-nucleosomes with micrococcal nuclease. Aliquots of digested chromatin samples were then immunoprecipitated with antibodies against H3K4me1, H3K4me3, and H3K9ac. Antibodies of which the specificities have been ascertained by the modENCODE project [36, 54] were obtained from Abcam, Inc., and Active Motif, Inc.

Enriched chromatin extracts were used to prepare bar-coded sequencing libraries. ChIP libraries of the four conditions from each replicate were pooled and sequenced in one 50 bp single read Illumina HiSeq2000 lane. ChIP sequences were aligned to the reference genome using Bowtie2-2.0.6 followed by peak calling using MACS1.4.2 (Model-based Analysis for ChIP-Seq) [47, 55]. Peak calls were merged across samples if the peak was present in more than one sample and the peak width bigger than 146 bp. Sequence counts were then summed for each peak, and normalized across samples by calculating Fragment Per Kilobase per Million reads mapped (FPKM), corrected for background using median FPKM of non-peak intervals in each sample and followed by quantile normalization across samples. We used background corrected normalized values of FPKM for each peak for differential modification analysis, as described above, using permutation with one-way ANOVA and FDR to correct for multiple-testing under dependency [51]. We used one pooled standard deviation for *post hoc* pairwise comparisons. Raw data can be accessed in the National Center for Biotechnology Information Sequence Read Archive (SRA) under accession number SRP048404.

### Gene ontology analysis

Gene ontology enrichment analyses for transcripts that changed expression after mating or aging was done using DAVID [56] with a cut-off for nominal statistical significance of *P* < 0.01.

### Confirmation of interactions between miRNAs and target genes

We obtained *UAS-mir-281-1*, *UAS-mir-286*, *UAS-mir-34*, *UAS-mir-92b*, *UAS-mir-988, mir-310* and the co-isogenic control lines from the Bloomington Stock Center. Females from each homozygous *UAS-mir* line and their progenitor control were crossed with males from a *ubiquitin-GAL4* driver line (*w*^*1118*^*: Ubi-Gal4*). We did not obtain viable offspring when *mir-281-1* or *mir-310* was overexpressed under the ubiquitin promoter. For the other crosses 4–6 day old virgin and mated F1 females were flash frozen for RNA extraction. We extracted total RNA from 10 female flies of each replicate with three biological replicates of each cross. RNAs were converted to cDNA using the iScript cDNA Synthesis Kit (Bio-Rad, Inc.). We used real time quantitative PCR on an ABI 7900HT qRT-PCR thermal cycler with 3 technical replicates for each sample and ABsolute qPCR SYBR Green Mixes (Thermo Scientific, Inc.) to evaluate the difference in expression of miRNA target genes between virgin and mated F1 females in controls and between virgin and mated F1 females that overexpress miRNAs (Table 3) using the following primers: CCCTGCTCCTTCTCCTTCTT and TGAACTTGAACCCCACCTTG for *Mlc1*, GGTCATCGTCTGTCCGATCT and CTAACTTCCTGGGCGACAAC for *dyl*, CACCCCAATTCACTCTGGTT and CCTTCAGCCGAAGATTGAAA for *Egfr*, GGAGACACAGCTCCGACAAT and ATTCTGGCTACCCGGCTTAT for *Vha68-1*, TTTCGGTGTCTTGTGTCTGG and AGGCAGTCGCTGTAGATGCT for *Osi7*, GTCCCTGATCGGCGATAATA and AACCCAGGAGCTTCTTCAACT for *CG15212*, TCCGAGTACTCTCCCTCCAG and TCTTGCTAGTCCAGCCACCT for *CG4000*, CAGTAGTGGAGCAGGCAGTG and ATGATAGGCCTCCTCCTTGG for *Pkcdelta*, GGGATAGGCTGGATTGGATT and CGTCGAGAGTAGCTCCGATT for *CG31869*, AGTAGTTGCGATGCCAATCC and AGGCGCTATAATTTCGATGC for *CG8303*, TGATCTTGGCAGTGGACTTG and AAGATTCTGCGCAAGAAGGA for *Prm*, CACTGGCATACCTTTGGTTG and TGGCAGTGACATTGATTCGT for *fln*, AGAATTGCCTTTTGCCACA and CCGACTACGATTCGAAAAGC for *Tusp*, ATTGCAGTGTGGTTGTTCCA and CCAGTTTGGTTGGAGACGAT for *CG34040*, GCGAAAGAGAGTCCGAGAGA and GGTTGTGTGCTGGTCAGTTG for *dan*, CATCGGCATCAGCATCTATC and GCATAGATGGATGGGTGGAC for *CG16867*, GTAATGACGGTGGCCTTTTC and CTTTGACCATCTTGGCCACT for *Gbeta76C*, AGGTCTTGGGCCTAGGTGTT and GAGGCCGATCCTGATGAATA for *prc*, CAAATCCTGCGCCTCATAGT and CACATCGAGTGCCTTGGATA for *CG15571*, GGTGGAGCTGGAGAACTACG and AGGTCTCTCCGTCGACATTC for *CG31086*, TCACCAGATTCCCGCTTATC and GTAACCACCGGATGATGAGG for *Ela* and ACTGAGAGCCAGGACTGGAA and CTTCCGGTTTCACAGATGGT for *vkg. Gapdh* expression (Primers: AGGCGTTTGTGACTTCTGGAA and TCTGGCCGTTGAGCATTTC) in each sample was used to normalize gene expression by mean normalization.

### Oviposition

We collected 5–7 day old-mated F1 control females and females overexpressing miRNAs under the ubiquitin-GAL4 promoter and counted the number of eggs laid by 5 females during 18 hours (4:00 pm-11:00 am). Ten replicates of 5 F1 females were tested from each cross. We used Dunnett’s test to assess variation of egg laying between the control and miRNA overexpression crosses.

## Electronic supplementary material

Additional file 1: **Differences in gene expression levels.** **Table S1.** Differences in gene expression levels between young and aged female flies. **Table S2.** Differences in gene expression levels between virgin and mated female flies. (XLSX 119 KB)

Additional file 2: Figure S1: Four-way Venn diagram that shows overlap among transcripts that change in abundance level after mating of young flies (green circle) and aged flies (red circle), and as a result of aging in virgin flies (blue circle) and mated flies (yellow circle). (TIFF 2 MB)

Additional file 3: Table S3: Genes that generate degraded fragments after mating. (DOCX 13 KB)

Additional file 4: **Changes in miRNA expression levels upon mating and aging.** (XLSX 16 KB)

Additional file 5: Figure S2: Four-way Venn diagram that shows overlap among miRNAs that change in abundance level after mating of young flies (green circle) and aged flies (red circle), and as a result of aging in virgin flies (blue circle) and mated flies (yellow circle). (TIFF 2 MB)

Additional file 6: Figure S3: Four-way Venn diagrams that show overlap among histone modifications that change in modification intensity after mating of young flies (green circle) and aged flies (red circle), and as a result of aging in virgin flies (blue circle) and mated flies (yellow circle). **(a)** H3K4me1 modification. **(b)** H3K4me3 modification. **(c)** H3K9ac modification. (TIFF 998 KB)

Additional file 7: **Changes in H3K4me1 modification.** **Table S4.** Changes in H3K4me1 modification between young and aged female flies. **Table S5.** Changes in H3K4me1 modification between virgin and mated young female flies. (XLSX 298 KB)

Additional file 8: **Changes in H3K4me3 modification.** **Table S6.** Changes in H3K4me3 modification between young and aged female flies. **Table S7.** Changes in H3K4me3 modification between virgin and mated female flies. (XLSX 610 KB)

Additional file 9: **Changes in H3K9ac modification.** **Table S8.** **(a)** Changes in H3K9ac modification between young and aged virgin female flies. **(b)** Changes in H3K9ac modification between young and aged mated female flies. **Table S9.** **(a)** Changes in H3K9ac modification between virgin and mated young female flies. **(b)** Changes in H3K9ac modification between virgin and mated aged female flies. (XLSX 715 KB)

Additional file 10: Figure S4: Three-way Venn diagrams that show overlap among H3K4me1 (blue circle), H3K4me3 (green circle) and H3K9ac (yellow circle) modifications that change in modification intensity after mating of young flies **(a)**, and aged flies **(b)**, and as a result of aging in virgin flies **(c)** and mated flies **(d)**. (TIFF 832 KB)

Additional file 11: **Histone modifications and associated genes that are response for mating-induced accelerated aging.** **Table S10.** Genes associated with H3K4me1 modification changes. **Table S11.** Genes associated with H3K4me3 modification changes. **Table S12.** Genes associated with H3K9ac modification changes. (XLSX 49 KB)

Additional file 12: **Genes identified from the DroID database that can interact with miRNA target genes which change expression after mating or upon aging.** **Table S13.** Genes that can interact with miRNA target genes which change expression in young flies after mating. **Table S14.** Genes that can interact with miRNA target genes which change expression in virgin flies upon aging. **Table S15.** Genes that can interact with miRNA target genes which change expression in mated flies upon aging. (XLSX 76 KB)
