# Supplementary figures and images for: Transcriptional and epigenetic responses to mating and aging in Drosophila melanogaster
Source: BMC Genomics. 2014 Oct 23;15(1):927. doi: 10.1186/1471-2164-15-927 (PMC4221674; doi:10.1186/1471-2164-15-927)

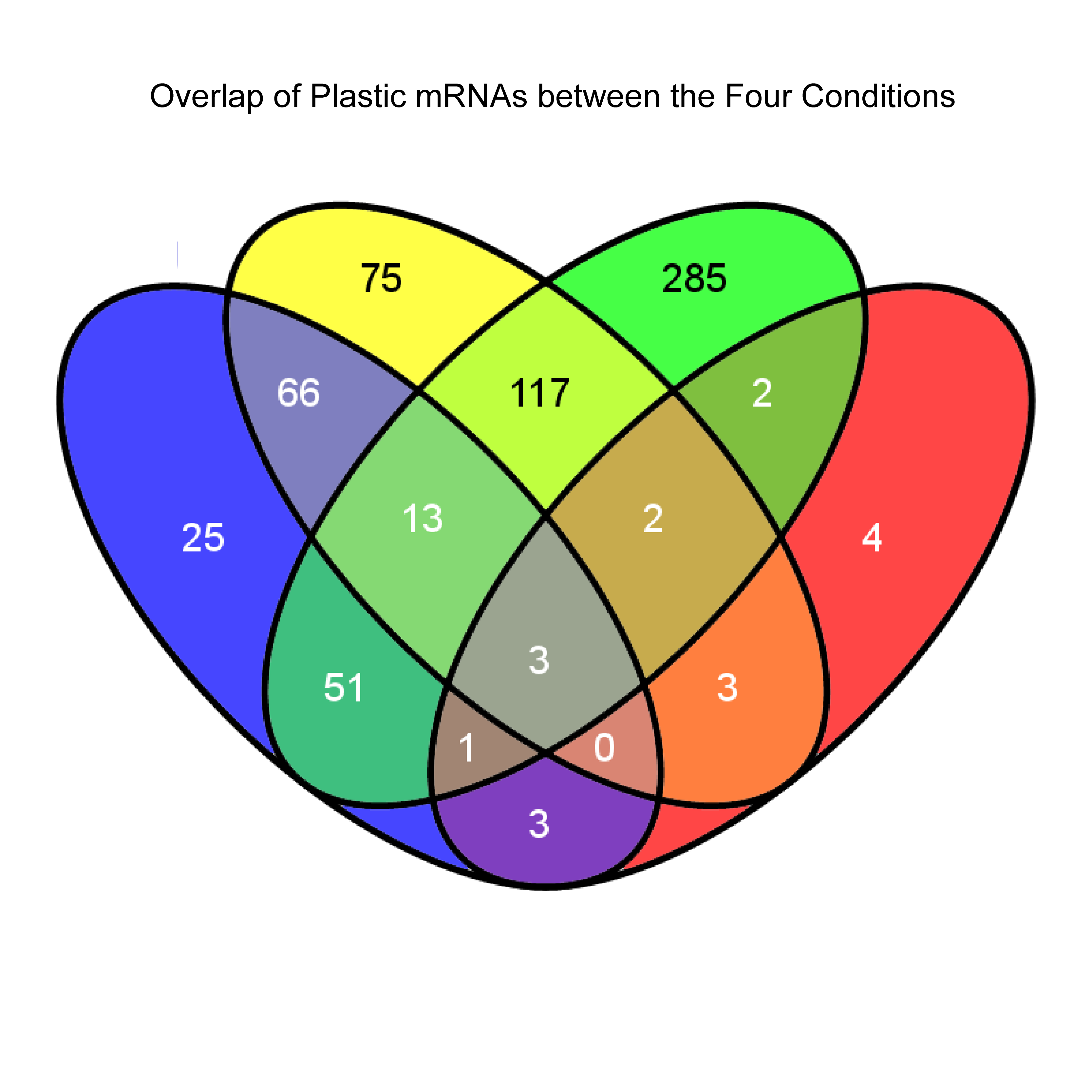

Supplement: Supplementary file 2 — Additional file 2: Figure S1: Four-way Venn diagram that shows overlap among transcripts that change in abundance level after mating of young flies (green circle) and aged flies (red circle), and as a result of aging in virgin flies (blue circle) and mated flies (yellow circle). (TIFF 2 MB) [file 12864_2014_6621_MOESM2_ESM.tiff]

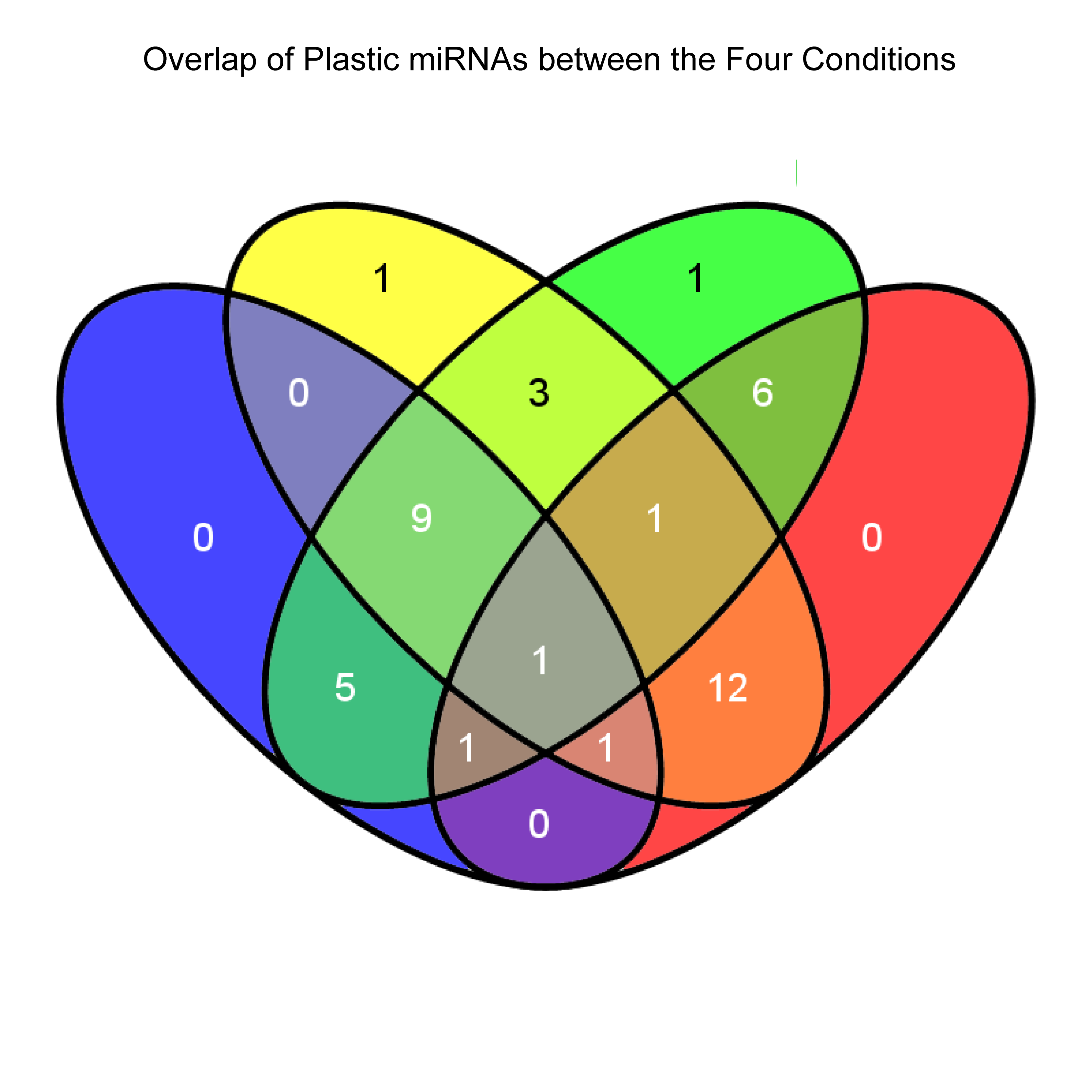

Supplement: Supplementary file 5 — Additional file 5: Figure S2: Four-way Venn diagram that shows overlap among miRNAs that change in abundance level after mating of young flies (green circle) and aged flies (red circle), and as a result of aging in virgin flies (blue circle) and mated flies (yellow circle). (TIFF 2 MB) [file 12864_2014_6621_MOESM5_ESM.tiff]

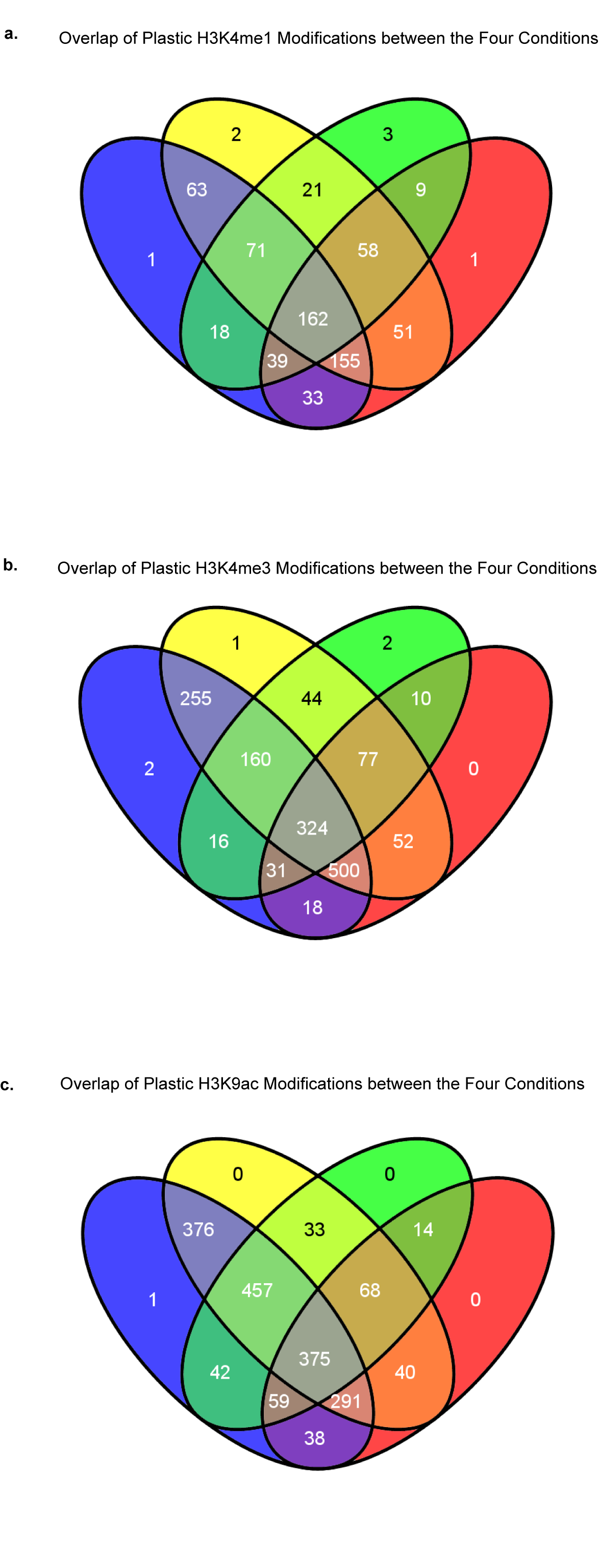

Supplement: Supplementary file 6 — Additional file 6: Figure S3: Four-way Venn diagrams that show overlap among histone modifications that change in modification intensity after mating of young flies (green circle) and aged flies (red circle), and as a result of aging in virgin flies (blue circle) and mated flies (yellow circle). (a) H3K4me1 modification. (b) H3K4me3 modification. (c) H3K9ac modification. (TIFF 998 KB) [file 12864_2014_6621_MOESM6_ESM.tiff]

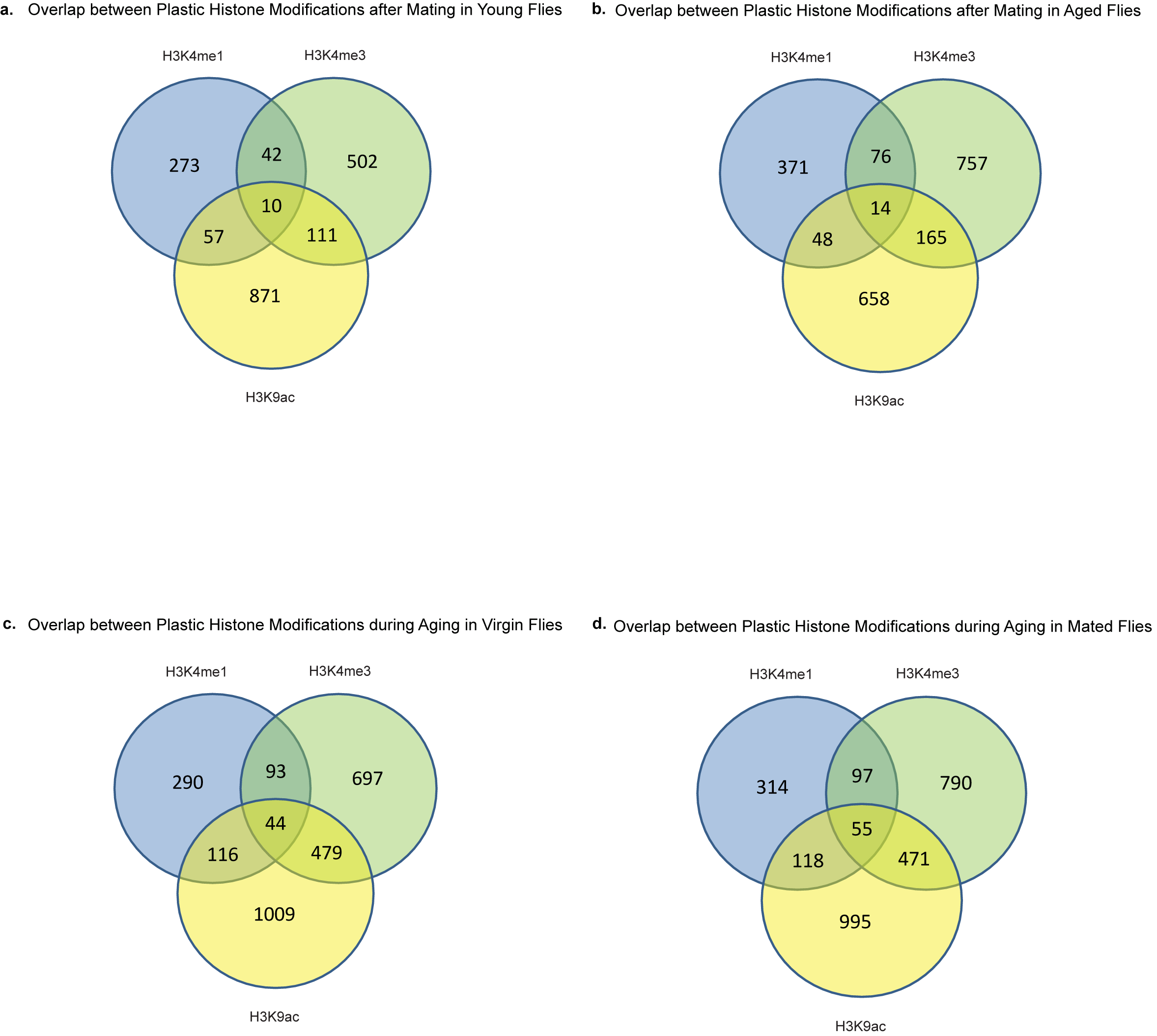

Supplement: Supplementary file 10 — Additional file 10: Figure S4: Three-way Venn diagrams that show overlap among H3K4me1 (blue circle), H3K4me3 (green circle) and H3K9ac (yellow circle) modifications that change in modification intensity after mating of young flies (a), and aged flies (b), and as a result of aging in virgin flies (c) and mated flies (d). (TIFF 832 KB) [file 12864_2014_6621_MOESM10_ESM.tiff]
