# Supplementary material for: Transcriptional and epigenetic responses to mating and aging in Drosophila melanogaster
Source: BMC Genomics. 2014 Oct 23;15(1):927. doi: 10.1186/1471-2164-15-927 (PMC4221674; doi:10.1186/1471-2164-15-927)
Supplement: Supplementary file 3 — Additional file 3: Table S3: Genes that generate degraded fragments after mating. (DOCX 13 KB) [file 12864_2014_6621_MOESM3_ESM.docx]

Additional file 3: Table S3: Genes that generate degraded fragments after mating.

| *betaTub85D* | *CG15730* | *CG43193* | *dysc* | *Myd88* | *RhoGEF3* |
| --- | --- | --- | --- | --- | --- |
| *bmm* | *CG15882* | *CG4788* | *EDTP* | *Na* | *Rip11* |
| *Ca-beta* | *CG1688* | *CG5404* | *elav* | *Nep4* | *ry* |
| *calypso* | *CG17260* | *CG5521* | *ERp60* | *nonC* | *salm* |
| *Ccp84Ad* | *CG17294* | *CG5681* | *f* | *Nop60B* | *Sam-S* |
| *ced-6* | *CG18619* | *CG5787* | *fred* | *Nopp140* | *sbb* |
| *Cenp-C* | *CG2186* | *CG5938* | *Frq2* | *Or13a* | *sd* |
| *CG11403* | *CG2650* | *CG5953* | *fs(1)Yb* | *Or45a* | *Sin3A* |
| *CG12171* | *CG30116* | *CG6040* | *Gef26* | *Osi17* | *Sod3* |
| *CG12213* | *CG30413* | *CG6432* | *Gfat1* | *osm-1* | *SsRbeta* |
| *CG12502* | *CG31100* | *CG6527* | *Gr39b* | *Osp* | *sti* |
| *CG12795* | *CG31797* | *CG6938* | *Gr93d* | *Pdf* | *stj* |
| *CG12920* | *CG32206* | *CG7265* | *Grip75* | *Pgd* | *Su(Tpl)* |
| *CG13516* | *CG33123* | *CG7461* | *Hel89B* | *Plc21C* | *TfIIEbeta* |
| *CG13606* | *CG33232* | *CG7786* | *Hsc70-3* | *pod1* | *thoc6* |
| *CG13870* | *CG33278* | *CG7927* | *Imp* | *Poly* | *TyrR* |
| *CG14143* | *CG33307* | *CG8331* | *Ir68b* | *Pps* | *unc-13-4A* |
| *CG14257* | *CG33510* | *CG8475* | *jvl* | *Prat* | *v(2)k05816* |
| *CG14329* | *CG33914* | *CG8534* | *kat80* | *Prc* | *VAChT* |
| *CG14694* | *CG33937* | *CG8858* | *Khc-73* | *Put* | *Vrp1* |
| *CG14841* | *CG34347* | *CG9062* | *Lip1* | *Qua* | *wrapper* |
| *CG14937* | *CG3557* | *CR42646* | *Lk* | *Rabex-5* | *Xrp1* |
| *CG15144* | *CG42399* | *Cyp12a4* | *LysE* | *Ranshi* |  |
| *CG15523* | *CG42564* | *Dhc36C* | *mia* | *Rbf2* |  |
| *CG15534* | *CG42817* | *Dic4* | *Mur89F* | *RhoGAP92B* |  |
